# Editorial: Mass spectrometry and new computer-based tools in plant science research

**DOI:** 10.3389/fpls.2026.1836465

**Published:** 2026-04-13

**Authors:** Letizia Bernardo, George V. Popescu, Candida Vannini

**Affiliations:** 1 Department of Agricultural and Environmental Science, University of Milan, Milan, Italy; 2 Institute for Genomics, Biocomputing and Biotechnology, Mississippi State University, Starkville, MS, United States; 3 Department of Biotechnology and Life Sciences, University of Insubria, Varese, Italy

**Keywords:** complex plant system, computational analysis, integration, mass spectrometry, omics

Mass spectrometry (MS) technologies have transformed plant science by enabling detailed characterization of biomolecules and investigation of biological processes and functions across multiple scales. Advances in analytical instrumentation, together with increasingly sophisticated computational tools, now allow researchers to identify and quantify proteins, metabolites, and other molecular components with unprecedented sensitivity and throughput. These developments have made MS-based approaches indispensable for investigating plant physiology, metabolism, stress responses, and interactions with pathogens and the environment, and quality assessment.

The Research Topic *“Mass Spectrometry and New Computer-Based Tools in Plant Science Research”* was launched to highlight methodological innovations and emerging applications that combine high-throughput MS technologies with advanced computational analysis. In particular, this Research Topic emphasizes the growing synergy between experimental mass spectrometry and computer-based tools, including bioinformatics pipelines, statistical frameworks, and machine learning approaches, to interpret complex molecular datasets and uncover biological mechanisms in plants. The eight contributions gathered in this Research Topic illustrate how integrative analytical strategies are advancing plant systems biology and providing new perspectives for crop improvement and plant biotechnology.

Integrated proteomic and metabolomic analyses, particularly revealed molecular differences between resistant and susceptible roots of chilli pepper (*Capsicum annuum*) during *Phytophthora capsici* infection, highlighting coordinated defense-related metabolic reprogramming. By following early and later infection stages, the study showed that resistance is associated with dynamic shifts in metabolite classes and protein abundance, identifying arachidonic acid (AA) as the most significant differentially abundant metabolite (Heng et al.).

Salinity tolerance, another major agricultural challenge, is examined in alfalfa through comparative transcriptomic and proteomic analyses of two genotypes with different responses to salt stress. The study identified pathways related to photosynthesis, secondary metabolism, signaling, lipid metabolism, oxidative processes, and stress defense, along with candidate genes that may contribute to improved tolerance. Importantly, the comparison between a more tolerant and a more sensitive genotype helped distinguish general stress responses from mechanisms more specifically associated with resilience (Hang et al.).

Fruit quality and diversity are further examined in a study investigating molecular traits among Italian sweet cherry (*Prunus avium*) accessions through integrated proteomic and metabolomic analysis. By combining MS-based metabolite profiling with protein identification, the authors uncovered molecular signatures (in particular flavonoid and anthocyanin levels) associated with biochemical diversity in cherry fruits (De Pascale et al.). The results underscore how MS-driven multi-omics approaches can help elucidate the biochemical foundations of fruit composition and potentially guide breeding strategies aimed at improving nutritional and sensory traits.

Another contribution explores the sensory attributes in cacao by performing a comparative proteomic analysis of unfermented cocoa beans from fine-flavor and bulk genotypes. Using high-resolution MS, the study identified protein differences linked to flavor-related biochemical pathways, providing insights into the molecular determinants of cacao quality for genotype selection and improvement (de Oliveira et al.).

Beyond crop quality and plant defense, MS technologies also provide valuable insights into fundamental aspects of plant metabolism and development. One contribution focuses on the biofuel crop pennycress (*Thlaspi arvense*), where combined transcriptomic and proteomic analyses were used to investigate the dynamics of oil body-associated proteins, including oleosins, seipins, caleosins, and stereolisins, OBAPs, and LDAPs during seed development (Luján et al.). The study highlights regulatory mechanisms controlling lipid storage and mobilization in seeds, offering insights relevant to the development of sustainable bioenergy crops.

In *Polygonatum odoratum*, targeted metabolomics and proteomics across developmental stages mapped the seasonal accumulation of bioactive compounds and linked specific proteins to flavonoid and steroidal saponin biosynthesis in winter and in spring, respectively. By identifying stage-specific patterns in both metabolites and protein expression, the study offers a framework for optimizing extraction strategies and for investigating the regulatory mechanisms underlying the accumulation of desirable compounds (Liang et al.).

A comparative proteomic analysis of immature xylem in *Eucalyptus urophylla × Eucalyptus grandis* examined wood formation across different ages and identified differentially abundant proteins associated with cell expansion, secondary wall biosynthesis, lignification, and programmed cell death, focusing on those subjected to post transcriptional and post translational modifications. The resulting protein-level view of secondary growth provides insights into potential regulatory mechanisms of key proteins involved in eucalyptus wood formation and constitutes a molecular foundation for future work on forest breeding, wood quality, and biomass production (Liu et al.).

Finally, this Research Topic also includes a computationally-driven investigation combining computational proteomics with molecular docking and simulation. In *Taphrina deformans*, the causal agent of peach leaf curl disease, a computational proteomics pipeline was used to prioritize essential proteins and identify glutamate-cysteine ligase as a promising antifungal target. Virtual screening, docking, and molecular dynamics simulations then linked that target to candidate commercial fungicides, demonstrating how in silico workflows can accelerate hypothesis generation and target validation in plant pathology (Ahmad et al.).

Taken together, the contributions in this Research Topic demonstrate the versatility of MS-based approaches for addressing diverse biological questions, spanning from plant–pathogen interactions and crop quality traits to seed metabolism and developmental processes. Mass spectrometry has become a central tool for uncovering molecular processes that shape plant physiology and adaptation.

A key theme emerging from these studies is the importance of integrating mass spectrometry with computational analysis. Modern MS instruments generate large and complex datasets that require advanced bioinformatics pipelines and statistical frameworks for interpretation. As illustrated across the contributions in this Research Topic, the integration of MS-based experimental workflows with data-driven computational methods provides a powerful framework for exploring plant molecular systems. Altogether, by increasing the use of multi-omics approaches, combining proteomics, metabolomics and transcriptomics data, researchers can build more comprehensive models of plant biological processes. Such integrative strategies allow scientists to connect gene expression patterns with protein abundance, metabolic fluxes, and physiological responses. The resulting systems-level perspectives are essential for understanding how plants respond to environmental stress, regulate metabolic pathways, and maintain cellular homeostasis.

In summary, the studies collected in this Research Topic highlight the growing impact of mass spectrometry and computational analysis in plant science research. By combining cutting-edge analytical technologies with innovative data analysis tools, these contributions provide new insights into plant molecular biology, moving beyond descriptive proteomics and metabolomics towards system-level investigations and demonstrate the potential of integrative approaches for addressing complex biological questions.

